# Development and Psychometric Validation of a Multidimensional Ecological Model-Based Awareness Scale for Patients with Stage 3–4 Chronic Kidney Disease

**DOI:** 10.3390/healthcare14070876

**Published:** 2026-03-28

**Authors:** Berrak Itır Aylı, Nüket Paksoy Erbaydar

**Affiliations:** 1 Institute of Health Sciences, Department of Public Health, Hacettepe University, 06100 Ankara, Türkiye; 2 Department of Public Health, Faculty of Medicine, Hacettepe University, 06100 Ankara, Türkiye

**Keywords:** chronic kidney disease, disease awareness, health literacy, patient-reported outcome, psychometric evaluation, scale development, scale validation, socioecological model

## Abstract

**Highlights:**

**What are the main findings?**

- A 34-item multidimensional CKD Awareness Scale (CKD-AS-34) was developed for stage 3–4 patients and demonstrated a stable seven-factor structure with excellent reliability (α = 0.884; ω = 0.889; ICC = 0.954).
- The scale captures CKD awareness across core socioecological levels (individual, interpersonal/institutional, community, and systemic), complemented by treatment adherence and social impact dimensions, with strong criterion validity against health literacy (r = 0.810).

**What are the implications of the main findings?**

- The CKD-AS-34 enables clinicians and researchers to identify specific awareness deficits across individual, interpersonal, community, systemic, and treatment-adherence domains.
- The scale provides a practical tool to guide targeted education, risk stratification, and public health interventions aimed at slowing CKD progression and improving patient outcomes.

**Abstract:**

**Background and Objectives:** Despite critically low levels of chronic kidney disease (CKD) awareness worldwide, there is no psychometrically validated instrument to comprehensively assess CKD awareness across socioecological levels. This study aimed to develop, psychometrically evaluate and validate a multidimensional awareness scale grounded in socioecological theory for patients with stage 3–4 CKD. **Materials and Methods:** This methodological study enrolled 908 stage 3–4 CKD patients. Scale development proceeded through systematic stages: comprehensive literature review, qualitative interviews (*n* = 15), expert panel evaluation (*n* = 25), and pilot testing. The initial 72-item pool was refined to 41 items (Content Validity Index = 0.912). The sample was randomly split for exploratory factor analysis (EFA; *n* = 454) and confirmatory factor analysis (CFA; *n* = 454). Psychometric evaluation encompassed internal consistency (Cronbach’s α, McDonald’s ω), test–retest reliability (*n* = 30; 4-week interval), convergent validity (average variance extracted [AVE], composite reliability [CR]), discriminant validity (Fornell–Larcker criterion), and criterion validity (correlation with Turkish Health Literacy Scale-32 [TSOY-32]). **Results:** EFA revealed a seven-factor structure with an acceptable explained variance of 43.8%. Following iterative item elimination based on communalities (h^2^ < 0.20) and factor loadings (λ < 0.30), CFA confirmed the final 34-item model with good fit (CFI = 0.972; RMSEA = 0.070 [90% CI: 0.067–0.074]). The factor structure captured awareness across core socioecological levels (Individual, Interpersonal/Institutional, Community, and Systemic), complemented by Treatment Adherence and Social Impact dimensions. Internal consistency coefficients were α = 0.884 and ω = 0.889 for the total scale. Test–retest reliability yielded an ICC of 0.954 (95% CI: 0.907–0.978). Convergent and discriminant validity were confirmed via composite reliability (CR: 0.740–0.953) and the Fornell–Larcker criterion. Criterion validity analysis revealed a significant correlation with TSOY-32 (r = 0.810, *p* < 0.001). **Conclusions:** The CKD Awareness Scale (CKD-AS-34) represents a novel, psychometrically validated, multidimensional awareness instrument for CKD. This scale enables clinicians to identify awareness deficits spanning individual to systemic levels, facilitating personalised patient education and targeted public health interventions.

## 1. Introduction

Chronic Kidney Disease (CKD) represents a major and growing global public health challenge characterised by rapidly increasing prevalence, substantial morbidity and high mortality. According to World Health Organisation data, non-communicable diseases account for 74% of global mortality, with CKD emerging as one of the fastest-growing contributors within this category [1,2]. CKD typically follows an insidious clinical course, progressing silently and remaining asymptomatic during early stages and consequently resulting in delayed diagnosis. As the disease progresses, it precipitates serious complications including cardiovascular disease, anaemia, mineral-bone disorders, metabolic acidosis, and ultimately end-stage kidney disease, profoundly diminishing quality of life while imposing a considerable strain on healthcare systems [3,4].

Recent epidemiological evidence underscores the escalating global burden of CKD. The 2023 Global Burden of Disease (GBD) Study estimated a global CKD prevalence of 14.2%, identifying CKD as one of the leading causes of disability-adjusted life years and mortality worldwide [2]. The 2011 CREDIT study in Turkey documented a national prevalence of 15.7%, whereas the 2023 GBD study estimated an age-standardised prevalence of 17%, substantially exceeding the global average [2,5]. Critically, future projections indicate that CKD prevalence will continue to rise [6]. Within this context, patients with stage 3–4 CKD represent a particularly critical group. These stages mark a pivotal period during which timely interventions, including medical management and lifestyle modification, can slow disease progression, delay renal replacement therapy, and improve both physical and psychological outcomes. Conversely, inadequate management during this phase often results in rapid deterioration and preventable mortality [3,7,8,9,10,11,12,13,14,15].

CKD awareness exerts multifaceted effects across multiple ecological levels, yet current evidence consistently demonstrates critically low awareness both among patients and within healthcare systems [16,17,18,19,20,21,22,23,24,25,26,27,28,29]. At the individual level, disease knowledge directly influences symptom recognition, treatment adherence, lifestyle modification, and healthcare-seeking behaviours [20,21,25]. However, studies have reported concerning findings: 69% of patients in the United States remain unaware of their kidney disease diagnosis [22]; 93% of participants in an Egyptian study believed in the effectiveness of alternative treatments for CKD [18]; and 56% of stage 3 to 5 CKD patients in the United States had no familiarity with kidney transplantation [23]. Results from Turkey reveal equally low awareness levels, with 92.5% of dialysis patients unable to define haemodialysis [28] and 24.5% of the population reporting regular use of non-prescribed medications [29]. Importantly, the awareness deficit extends beyond patients into healthcare services, where substantial numbers of patients experience missed diagnoses and delayed nephrology referrals [30,31,32,33,34]. The multinational REVEAL-CKD study revealed that 95% of stage 3 CKD patients in France, 84% in Germany, and 92% in Japan were not recorded as having CKD despite glomerular filtration rates below 60 mL/min/1.73 m^2^, while in the United States, 64% of patients experienced delayed nephrology referrals [32,34].

This dual deficit, late recognition within healthcare systems and low patient awareness, creates a bidirectional burden that accelerates disease progression and amplifies preventable complications. This manifests through multiple pathways: inadequate lifestyle modification following delayed diagnosis and late presentation, non-adherence to pharmacological and dietary recommendations, accelerated disease progression, frequent complications, increased cardiovascular events, emergency and unplanned RRT initiation, and escalating healthcare system burden and costs.

Furthermore, disease awareness transcends mere cognitive understanding; it constitutes a complex social, psychological, and structural phenomenon. Health behaviour theories, including Bronfenbrenner’s ecological systems theory and McLeroy’s socioecological model, emphasise that health-related behaviours and outcomes are shaped by interacting influences across multiple levels, including interpersonal relationships, healthcare institutions, community contexts, and broader health system structures [35,36]. Within this theoretical framework, CKD awareness is influenced not only by individual knowledge and symptom recognition, but also by family and social support, patient–provider communication, healthcare accessibility, community beliefs, and systemic characteristics of health services. Therefore, a holistic and multilevel approach to evaluating CKD awareness is imperative. A psychometrically validated measurement tool capable of comprehensively and multidimensionally assessing CKD awareness constitutes a significant methodological gap in the existing literature. Although numerous awareness and self-management scales with robust psychometric properties have been developed for other chronic conditions—including diabetes, hypertension, asthma, and heart failure—no valid and reliable multidimensional scale exists for CKD.

Stage 3–4 CKD patients represent a strategically important population for the development of a comprehensive awareness assessment tool. Measuring awareness during this intervention-sensitive period may inform targeted educational strategies, improve patient engagement, and support more effective public health and clinical interventions.

To date, no psychometrically validated, multidimensional instrument exists for assessing CKD awareness across socioecological levels. This study aimed to address this methodological gap by developing and psychometrically validating a multidimensional measurement instrument to comprehensively evaluate disease awareness among stage 3–4 CKD patients in Türkiye, grounded in the socioecological model framework. The instrument was designed to capture awareness not only at the individual level, but also in relation to social support, interactions with healthcare services, community-level perceptions, treatment adherence, and the broader social impact of living with CKD.

## 2. Materials and Methods

### 2.1. Study Type and Design

This methodological study was conducted to develop a multidimensional awareness scale for patients with stage 3–4 chronic kidney disease (CKD) and to evaluate its psychometric properties. The scale development process followed internationally accepted methodological guidelines, including the theoretical framework specification, the COSMIN (COnsensus-based Standards for the selection of health Measurement INstruments) framework for patient-reported outcome measures, item generation, expert review, pilot testing, large-sample administration, and psychometric evaluation [37,38,39].

### 2.2. Theoretical Framework

The scale was grounded in Bronfenbrenner’s ecological systems theory and operationalised using McLeroy et al.’s socioecological model of health behaviour [35,36]. Accordingly, CKD awareness was conceptualised as a multidimensional construct influenced by factors at individual, interpersonal/institutional, community, and broader health system levels. This framework informed item development and factor structure expectations.

### 2.3. Study Population and Sample

The study population comprised patients with stage 3–4 CKD attending the Nephrology Clinic of Ankara Etlik City Hospital (Etlik Şehir Hastanesi), one of Türkiye’s largest tertiary public hospitals. Inclusion criteria were age ≥ 18 years, confirmed stage 3–4 CKD diagnosis according to KDIGO criteria, adequate Turkish language proficiency, and provision of informed consent. Patients with cognitive impairment due to dementia, Alzheimer’s disease, or other neurological conditions, communication barriers, previous kidney transplantation, or refusal to participate were excluded.

Sample size was determined based on recommendations for factor analysis and scale development, including participant-to-item ratios and minimum sample requirements for exploratory and confirmatory factor analysis [40,41,42,43]. Of 1000 eligible patients invited, 908 completed the questionnaire. To avoid cross-validation bias, the total sample was randomly split into two equal subsamples for exploratory factor analysis (EFA; *n* = 454) and confirmatory factor analysis (CFA; *n* = 454). An additional subsample (*n* = 30) was used for test–retest reliability assessment at a 4-week interval.

### 2.4. Scale Development Process

Item generation was informed by a systematic literature review, qualitative interviews with 15 CKD patients, and expert panel evaluation. Literature searches were conducted across PubMed, Web of Science, Scopus, Google Scholar, and Turkish Medline using predefined keywords related to CKD awareness, health literacy, and socioecological models. An initial pool of 72 items was generated.

**Figure 1:**
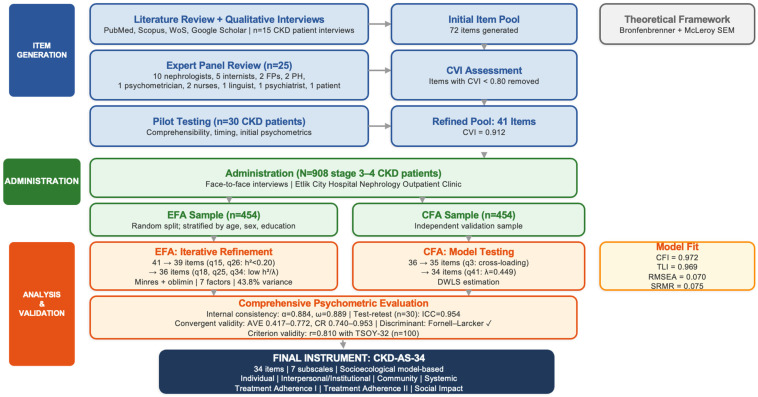
Overview of the CKD-AS-34 scale development and validation process.

A multidisciplinary expert panel (*n* = 25) comprising nephrologists (*n* = 10), internal medicine specialists (*n* = 5), family physicians (*n* = 2), public health specialists (*n* = 2), a psychometrician/scale development expert (*n* = 1), nephrology nurses (*n* = 2), a linguist (*n* = 1), a psychiatrist (*n* = 1), and a patient representative with stage 3–4 CKD (*n* = 1) reviewed all items for relevance and clarity. Each expert independently rated every item as ‘appropriate’, ‘requires modification’, or ‘should be removed’ and assessed whether items adequately represented their intended theoretical dimension. Content validity was assessed using the Content Validity Index (CVI), and items with CVI < 0.80 were removed. Following expert review and pilot testing with 30 patients, the item pool was refined to 41 items (CVI = 0.912). An overview of the complete scale development and validation process is presented in Figure 1.

### 2.5. Data Collection Process

Data were collected through face-to-face interviews conducted between September and December 2025 in a nephrology outpatient clinic. This approach was chosen to minimise missing data, accommodate patients with limited literacy or visual impairment, and ensure accurate item comprehension. Questionnaire completion required approximately 10–15 min.

Face-to-face interviews were conducted by the first author (B.I.A.). All participants were patients with confirmed stage 3–4 CKD already under regular nephrology follow-up at the clinic. Interviews were conducted in the outpatient clinic waiting area prior to scheduled nephrology appointments. This approach ensured that responses reflected patients’ baseline awareness rather than being influenced by information received during the consultation.

### 2.6. Statistical Analysis

All analyses were performed using R (version 4.5.1) on R Studio (version 2025.05.1). Item distributions were examined using descriptive statistics (mean, SD, median, skewness, kurtosis, floor and ceiling effects) [38]. Sampling adequacy was assessed with the Kaiser–Meyer–Olkin test and Bartlett’s test of sphericity. EFA was conducted using minimum residual extraction with oblimin rotation. Factor retention was guided by parallel analysis (1000 Monte Carlo simulations), scree plot inspection, and Kaiser criterion (eigenvalue > 1) [41,42,43]. Items with factor loadings < 0.30 or communalities < 0.20 were removed.

CFA was performed using diagonally weighted least squares estimation. Model fit was evaluated using standard indices (CFI, TLI, RMSEA, SRMR) [42,43,44]. Internal consistency was assessed using Cronbach’s alpha and McDonald’s omega. Convergent validity was examined using average variance extracted and composite reliability, while discriminant validity was assessed using the Fornell–Larcker criterion [45]. Test–retest reliability was evaluated using intraclass correlation coefficients. Criterion validity was examined via correlation with the Turkish Health Literacy Scale-32 in a subsample of 100 patients [46]. A subsample of 100 patients provides greater than 99% statistical power to detect the observed correlation of r = 0.810 and exceeds 80% power for correlations as low as r = 0.28 at α = 0.05.

### 2.7. Scale Scoring

The final CKD Awareness Scale consists of 34 items across seven subscales, rated on a five-point Likert scale. Subscale and total scores were calculated as mean item scores, with higher values indicating greater awareness. Negatively worded items were reverse-coded prior to score calculation (6 minus the original item score). No cut-off values were defined. Two response formats were applied; this approach, using agreement anchors (items 1–21) and frequency anchors (items 22–34), was intentional and theoretically motivated. Awareness perceptions are optimally captured through agreement scales that assess cognitive appraisal, whereas treatment adherence behaviours require frequency anchors that reflect actual behavioural patterns. This dual-format approach is consistent with established multidimensional instruments that combine attitudinal and behavioural item formats within a single scale.

The final Turkish version of the scale, its English translation for research purposes, and the detailed scoring rubric are provided in the Supplementary Materials.

### 2.8. Ethical Approval

Ethical approval was granted by Hacettepe University Health Sciences Research Ethics Committee (approval number: 2025/15-29, date: 22 July 2025). Written informed consent was obtained from all participants, and the study was conducted in accordance with the Declaration of Helsinki.

## 3. Results

### 3.1. Expert Panel and Item Pool Review

The initial 72-item pool underwent review by 25 experts; following elimination of items with CVI < 0.80, 41 items were retained, yielding a final CVI of 0.912. These items were initially distributed across theoretically defined domains aligned with the socioecological framework.

### 3.2. Descriptive Statistics and Item Characteristics

A total of 908 patients with stage 3–4 CKD completed the questionnaire. The socio-demographic and clinical characteristics of the study population are presented in Table 1. The mean age was 57.6 ± 17.2 years (range: 18–94); 54.1% were female. The majority were married (64.8%), and education levels ranged from illiterate (1.1%) to university-educated (20.0%). Retired individuals constituted the largest employment group (42.5%). Hypertension (61.0%) and diabetes mellitus (41.9%) were the most prevalent comorbidities.

Item means ranged from 1.44 to 4.41. All items exhibited skewness values below |2| and kurtosis values below |4|, confirming suitability for factor analysis (Table S1). No significant floor or ceiling effects were detected. Overall the KMO was 0.716, and Bartlett’s sphericity test was significant (χ^2^ (820) = 23,166.4, *p* < 0.001).

### 3.3. Initial Reliability Analysis (41-Item Form)

The initial 41-item scale demonstrated strong overall internal consistency (Cronbach’s α = 0.895; McDonald’s ω = 0.900). Most theoretically defined subscales exhibited acceptable reliability; however, the institutional awareness subscale showed relatively low internal consistency (α = 0.559), suggesting potential structural instability. This finding supported the need for exploratory factor analysis to empirically examine the underlying factor structure (Table 2).

### 3.4. Exploratory Factor Analysis

Exploratory factor analysis was conducted to empirically examine the underlying structure of the initial 41-item scale. Parallel analysis and scree plot inspection consistently supported a seven-factor solution, which remained stable throughout iterative item refinement. Analyses were performed using minimum residual extraction with oblimin rotation, given the expected correlations among dimensions.

The initial seven-factor model for the 41-item pool demonstrated acceptable fit (RMSEA = 0.049, 90% CI: 0.046–0.053; RMSR = 0.030; TLI = 0.863) and explained 40.4% of the total variance. Based on communality, factor loading, and item relevance criteria, items were removed in a stepwise manner. Questions 15, 18, 25 (blood pressure monitoring), 26 (blood glucose monitoring), and 32 were removed due to low communality, insufficient factor loadings, or high complexity values. The complete EFA item summary, including factor assignments, primary loadings, communalities, and retention decisions for all 41 items, is presented in Supplementary Table S2.

**Figure 2:**
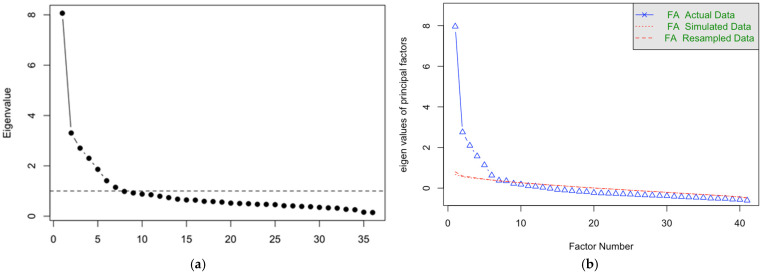
(**a**) Scree plot. (**b**) Parallel analysis.

The final EFA solution comprised 36 items across seven factors, explaining 43.8% of the total variance, with satisfactory model fit (RMSEA = 0.049; TLI = 0.885). Factor loadings and communalities fell within acceptable ranges. Observed eigenvalues were compared with simulated eigenvalues from parallel analysis (1000 Monte Carlo iterations) (Figure 2a,b). Parallel analysis supported a six-factor solution; however, the seventh factor (observed eigenvalue = 1.227, exceeding the Kaiser criterion of 1.0) was retained based on superior model fit of the seven-factor over the six-factor solution (TLI: 0.863 vs. 0.846; RMSEA: 0.049 vs. 0.052), theoretical alignment with the socioecological framework, and improved interpretability. The full eigenvalue comparison and model fit indices of alternative models are presented in Supplementary Tables S3 and S5.

Factor loadings ranged from 0.33 to 0.87, communalities from 0.23 to 0.78 for items; eigenvalues and explained variances of subscales are shown in Table 3.

### 3.5. Confirmatory Factor Analysis

The 36-item, seven-factor structure identified through EFA was subsequently tested using confirmatory factor analysis in an independent subsample. The initial CFA model demonstrated acceptable fit; however, modification indices suggested localised misfit attributable to cross-loading residuals (Table 4).

Following the removal of two items with problematic loadings (q3, EFA: h^2^ = 0.29, λ = 0.38) and residual correlations (q41, λ = 0.449), the final 34-item model achieved good overall fit (CFI = 0.972; TLI = 0.969; RMSEA = 0.070; SRMR = 0.075). Four items exhibited standardised CFA loadings between 0.538 and 0.621 (q4 = 0.538, q24 = 0.572, q23 = 0.610, q36 = 0.621), falling below the preferred 0.70 threshold while remaining above the minimum 0.50 criterion. These items were not removed because of their theoretical importance, acceptable loading levels, and the absence of cross-loading concerns. All standardised factor loadings were statistically significant and largely exceeded recommended thresholds, supporting the adequacy of the factor structure.

Final seven-factor structure:

- Factor 1—Individual Awareness (6 items: q1, q2, q6, q7, q8, q16)
- Factor 2—Interpersonal/Institutional Awareness (8 items: q4, q5, q9–q14)
- Factor 3—Community Awareness (3 items: q17, q19, q20)
- Factor 4—Systemic Awareness (4 items: q21–q24)
- Factor 5—Treatment Adherence I (4 items: q27-q29, q31)
- Factor 6—Treatment Adherence II (4 items: q30, q32, q33, q35)
- Factor 7—Social Impact (5 items: q36–q40).

**Figure 3:**
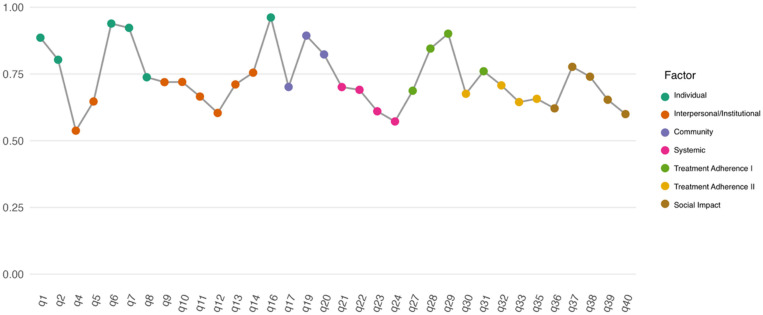
Standardised factor loadings of the 34-item final model.

Standardised factor loadings ranged from 0.538 to 0.962, with 29 of 34 items exceeding λ > 0.60 (Figure 3, Table S4).

### 3.6. Alternative Model Comparisons

To further evaluate construct validity, the empirical seven-factor model was compared with theoretically plausible alternative models, including a theoretical seven-factor model, a five-factor ecological model, a three-factor ecological model, and a single-factor model (Table S5). Because the alternative models tested were not hierarchically nested, each involving different item-to-factor assignments rather than parameter constraints within the same structure, formal chi-square difference testing was not applicable. Model selection was therefore based on the comparison of absolute and incremental fit indices (CFI, TLI, RMSEA, SRMR). The empirical seven-factor model demonstrated superior fit across all indices, confirming the multidimensional nature of CKD awareness and supporting the proposed factor structure.

**Figure 4:**
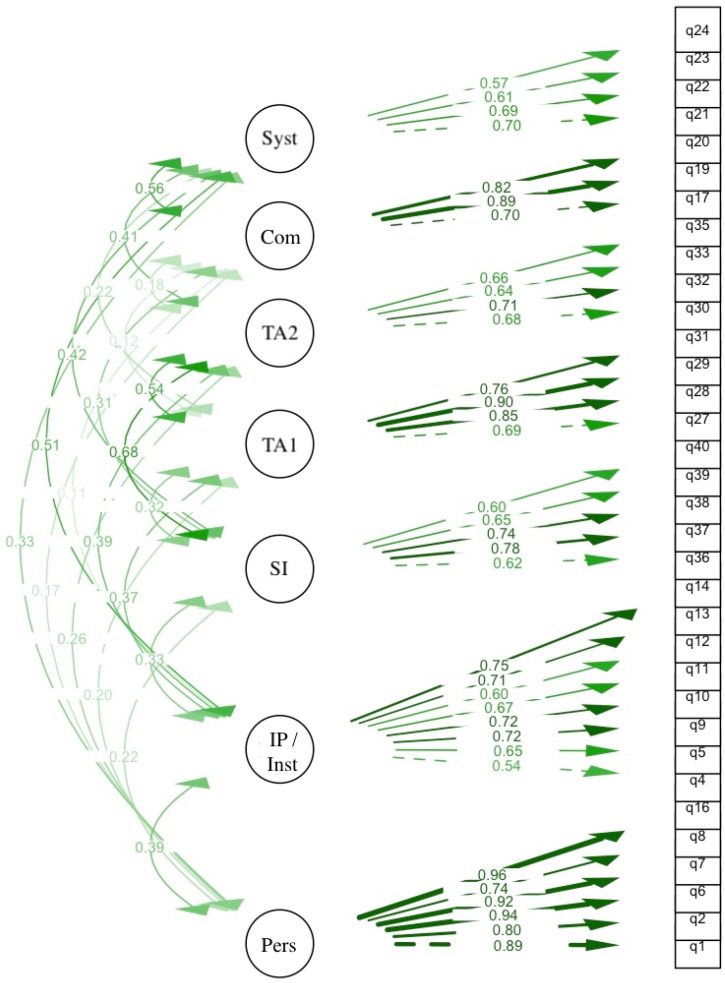
Confirmatory factor analysis diagram of the final model (7 empirical factors, 34 items). Syst = Systemic Awareness; Com = Community Awareness; TA2 = Treatment Adherence II; TA1 = Treatment Adherence I; SI = Social Impact; IP/Inst = Interpersonal/Institutional Awareness; Individual = Individual Awareness. Single-headed arrows represent standardised factor loadings; double-headed arrows represent inter-factor correlations.

The factor loadings of the empirical 7-factor model have been illustrated in Figure 4. All items exhibit factor loadings between 0.54 and 0.96, with interfactor correlations (shown as green lines) remaining between 0.11 and 0.68. The strongest correlations were noted between social impact and treatment adherence II (r = 0.68), systemic and community (r = 0.56), and treatment adherence I and II (r = 0.54).

### 3.7. Convergent and Discriminant Validity

AVE values ranged from 0.417 (Systemic) to 0.772 (Individual), while CR values ranged from 0.740 (Systemic) to 0.953 (Individual) (Table 5).

Inter-factor correlations ranged from 0.11 to 0.68. The highest correlation emerged between Social Impact and Treatment Adherence II (r = 0.68)—reflecting a theoretically meaningful relationship between social consequences of disease and adherence behaviours. Systemic-Community (r = 0.56) and Interpersonal/Institutional-Systemic (r = 0.51) correlations were moderate-high, indicating related yet distinct constructs. Critically, all √AVE values exceeded corresponding inter-factor correlations, satisfying the Fornell-Larcker criterion and confirming discriminant validity (Table 6).

### 3.8. Final Model Reliability

The final 34-item scale exhibited excellent overall internal consistency (α = 0.884; ω = 0.889). Subscale reliabilities have been presented in Table 7.

### 3.9. Test–Retest Reliability

In the test–retest subsample (*n* = 30, 4-week interval), item-level Pearson correlations ranged from 0.779 to 0.969, and the total scale demonstrated excellent stability (r = 0.976; ICC = 0.954 [95% CI: 0.907–0.978]) (Table 8). Bland–Altman analysis revealed no systematic bias between the two measurements. All participants maintained stable clinical status during the 4-week interval (no hospitalisations, no eGFR change exceeding 10%, no treatment modifications), as confirmed by medical record review. Paired-sample *t*-tests confirmed no statistically significant differences between Time 1 and Time 2 for either total scores or any subscale scores (all *p* > 0.05), consistent with the Bland–Altman findings.

### 3.10. Criterion Validity

In the subsample (*n* = 100), the CKD-AS-34 total score demonstrated a strong correlation with the TSOY-32 total score (r = 0.810, *p* < 0.001) supporting criterion validity.

## 4. Discussion

This study developed and comprehensively evaluated the psychometric properties of a multidimensional awareness scale grounded in ecological model theory for stage 3–4 CKD patients in Türkiye. Anchored in ecological systems theory, this 34-item scale comprehensively assesses disease awareness across dimensions spanning individual knowledge to systemic understanding as well as treatment adherence and the social impact of disease. The instrument demonstrated excellent factorial validity, strong reliability, and meaningful associations with health literacy, establishing itself as a valuable tool for clinical assessment, research applications, and public health programme evaluation.

### 4.1. Factor Structure and Theoretical Alignment

The emergence of a seven-factor structure corroborates the theoretical premise that CKD awareness constitutes a multidimensional construct transcending simple disease knowledge. The empirical model’s superiority over unidimensional and reduced ecological models highlights that awareness cannot be adequately captured through unidimensional or simplified assessments. Grounded in Bronfenbrenner’s ecological systems theory and McLeroy et al.’s socioecological model, the observed factor structure aligns closely with theoretical expectations and reflects the complex pathways through which awareness is shaped [35,36].

The merging of Interpersonal and Institutional dimensions into a single factor reflects the proximal social and healthcare environment of CKD patients in the Turkish context, in which family support and institutional healthcare encounters function as closely interacting microsystems. Within this context, family members frequently accompany patients to nephrology appointments, participate in dietary planning, and mediate communication with healthcare providers, creating a functionally integrated proximal environment consistent with Bronfenbrenner’s concept of mesosystem interactions between microsystems [35]. This empirical merger is clinically meaningful: patients’ perceived support from family and from healthcare providers are not experienced as separate ecological levels but as interconnected sources of proximal influence on awareness and engagement.

The bifurcation of Treatment Adherence into two empirically distinct factors, one emphasising CKD-specific dietary compliance (Treatment Adherence I: salt, protein, potassium, phosphorus restriction) and the other encompassing broader health management behaviours (Treatment Adherence II: medication adherence, fluid monitoring, avoidance of nephrotoxic agents, appointment attendance), reflects a well-established distinction in the chronic disease self-management literature. Dietary adherence in CKD involves highly specific nutritional restrictions requiring specialised knowledge and daily behavioural regulation, which are behaviourally and motivationally distinct from medication-taking and appointment-keeping behaviours [47]. This dietary–non-dietary distinction has been documented in other chronic conditions and is consistent with self-management frameworks that differentiate between disease-specific lifestyle modifications and general healthcare engagement behaviours [48]. Future research could explore whether method factors contribute to this bifurcation; however, the clinical utility of distinguishing dietary from non-dietary adherence supports the retention of two separate subscales.

Notably, Community Awareness emerged as a relatively independent dimension, exhibiting strong within-factor loadings but weaker associations with total awareness scores. This finding suggests that patients’ perceptions of societal understanding and stigma related to CKD may operate independently from their personal knowledge or clinical engagement, underscoring the need for community-level awareness interventions alongside individual education.

Inter-factor correlations ranged from 0.11 to 0.68, indicating that factors represent related yet distinguishable constructs. The highest correlation emerged between Social Impact and Treatment Adherence II (r = 0.68). This finding is theoretically meaningful, as a robust relationship is anticipated between disease impact on social life and treatment adherence behaviours, and difficulties in social functioning attributable to disease can substantially affect individuals’ adherence to treatment regimens [49].

These inter-factor relationships further underscore a key distinction between the CKD-AS-34 and existing instruments. Current tools for assessing CKD-related knowledge are limited in scope: the Kidney Disease Knowledge Survey [22] employs a unidimensional true/false format that captures only factual knowledge, while the Perceived Kidney Disease Knowledge Survey [23] assesses self-rated familiarity with treatment modalities alone. Neither instrument addresses the interpersonal, institutional, community, or systemic dimensions that shape how patients experience and act upon their disease knowledge. General health literacy tools such as the TSOY-32 [46], though correlated with the CKD-AS-34 (r = 0.810), similarly lack disease-specific and contextual content. By simultaneously capturing awareness across these socioecological levels alongside treatment adherence and social impact, the CKD-AS-34 provides a more clinically actionable assessment that can identify not only what patients know, but also the environmental and behavioural factors that determine whether that knowledge translates into effective self-management.

### 4.2. Item Elimination Process and Scale Optimisation

The systematic reduction from 72 initial items to 34 final items followed evidence-based psychometric criteria. Particularly noteworthy was the elimination of blood pressure measurement (q25) and blood glucose monitoring (q26) items, which exhibited low communalities, insufficient factor loadings, and structural heterogeneity. This finding reflects that self-monitoring behaviours for hypertension and diabetes, common CKD comorbidities with established public awareness, represent distinct constructs from CKD-specific awareness. Notable floor effects for items assessing GFR knowledge (q2; 51.8% scoring 1) and community awareness activities (q19: 54.6%; q20: 52.2%) corroborate the literature’s consistent finding of critically low CKD awareness at both individual and community levels. These distributional patterns reflect genuine population-level deficits in awareness. Whereas the ceiling effect for medication adherence (q30; 57.8% scoring 5) reflects a well-documented pattern in self-reported adherence measures across chronic diseases. These items were retained because they represent clinically essential content: the ability to detect low GFR awareness and community-level awareness gaps is precisely the scale’s intended function.

The progressive improvement in model fit indices, from initial CFI = 0.928 to final CFI = 0.972, demonstrates that item elimination strengthened rather than compromised structural validity. Each removal decision was predicated on empirical evidence rather than theoretical justification alone, ensuring that retained items optimally represent their theoretical constructs. While the upper bound of the RMSEA 90% confidence interval (0.074) approaches the conventional 0.08 threshold, this is consistent with the expected behaviour of RMSEA in larger samples where even minor misspecifications are detected. Importantly, all four fit indices converge on adequate-to-good fit: CFI (0.972) and TLI (0.969) exceed the stringent 0.95 criterion, and SRMR (0.075) remains well below 0.08, collectively confirming that the model provides a good representation of the data [43,44,45].

### 4.3. Reliability Assessment

Overall internal consistency (α = 0.884; ω = 0.889) positions the CKD-AS-34 among well-performing psychometric instruments. The close concordance between alpha and omega coefficients indicates that the scale largely satisfies tau-equivalence assumptions, with McDonald’s omega providing additional assurance for potentially heterogeneous structures.

The relatively lower alpha values for Systemic Awareness (α = 0.660) and Treatment Adherence II (α = 0.656) warrant contextual interpretation. These dimensions inherently encompass diverse content; Systemic Awareness addresses healthcare access, medication pricing, transportation, and health information policies; Treatment Adherence II covers medication use, fluid restriction, avoidance of unnecessary medications, and appointment adherence. Cortina [50] noted that lower alpha values are anticipated for heterogeneous constructs and that such dimensions should be supported by omega and composite reliability. Indeed, the omega (ω = 0.669 and ω = 0.697) and CR (0.740 and 0.767) values for these subscales fall within acceptable ranges. Additionally, test–retest ICC values for these subscales (0.904, 0.883) are excellent, confirming temporal stability despite content heterogeneity.

Additionally, from a clinical perspective, the Systemic Awareness and Treatment Adherence II subscales capture inherently heterogeneous behavioural and perceptual content that is clinically indispensable. Systemic Awareness items span healthcare access, medication pricing, transportation barriers, and health information policies; domains that cannot be collapsed without losing clinically meaningful information about distinct barriers. Treatment Adherence II encompasses medication adherence, fluid restriction, avoidance of unnecessary medications, and appointment attendance; each representing a different facet of active health management. The acceptable McDonald’s ω values (0.669 and 0.697), CR values exceeding 0.74, and excellent test–retest ICC values (0.904 and 0.883) collectively confirm that these subscales provide stable and reliable measurement despite the breadth of their content. These subscale scores remain clinically interpretable and may prove particularly useful for identifying specific intervention targets, as a patient scoring low on Systemic Awareness but high on Individual Awareness would benefit from healthcare navigation support rather than disease education.

Average variance extracted (AVE) values for four subscales (Systemic Awareness: 0.417; Treatment Adherence II: 0.451; Interpersonal/Institutional: 0.453; Social Impact: 0.465) fell below the conventional 0.50 threshold. However, Fornell and Larcker (1981) [45] noted that AVE is a conservative measure and that composite reliability (CR) exceeding 0.60 alone can provide sufficient evidence of convergent validity when construct heterogeneity is expected. All subscales demonstrated CR values above 0.74, and the Fornell–Larcker criterion for discriminant validity was satisfied across all factor pairs (Table 5). Nevertheless, AVE values below 0.50 for Systemic Awareness and Treatment Adherence II indicate that these subscales capture a relatively higher proportion of error variance relative to construct-explained variance. While composite reliability values and the Fornell–Larcker criterion confirm acceptable convergent and discriminant validity, future refinement of these subscales, through item revision, expansion, or rewording, may further enhance their measurement precision. This consideration is particularly relevant for Systemic Awareness, which had the lowest AVE (0.417) among all subscales.

The total explained variance of 43.8% is consistent with expectations for social-behavioural scales measuring complex, multidimensional constructs. Worthington and Whittaker [43] note that 40–60% explained variance is typical in such instruments, and the seven-factor structure was confirmed by CFA with good fit indices, providing stronger evidence of structural validity than explained variance percentage alone.

The strong criterion validity correlation with the TSOY-32 (r = 0.810) confirms the expected conceptual relationship between health literacy and disease awareness. However, the CKD-AS-34 captures dimensions entirely absent from health literacy instruments, including interpersonal and institutional support, community-level awareness, systemic healthcare perceptions, and social impact. The confirmed seven-factor structure and the superiority of the multidimensional model over a single-factor solution (Table S5) demonstrate that the CKD-AS-34 measures substantively more than health literacy alone. Health literacy may be understood as a foundational capacity that enables disease awareness, but awareness itself, as conceptualised within the socioecological framework, encompasses the contextual, relational, and experiential dimensions captured by the additional subscales.

### 4.4. Clinical and Policy Implications

The multidimensional structure of the CKD-AS-34 enables nuanced assessment of awareness deficits across ecological levels. Clinicians may identify patients with adequate individual knowledge but limited systemic or interpersonal awareness, guiding tailored interventions such as family engagement or healthcare navigation support. At the policy level, Systemic and Community Awareness scores may inform the design and evaluation of public awareness campaigns, healthcare access initiatives, and national CKD control strategies. In Türkiye, the Ministry of Health’s General Directorate of Public Health recently launched the Türkiye Kidney Diseases Prevention and Control Programme (2025–2030), which encompasses primary care strengthening through routine screening at family medicine level, optimisation of diabetes and hypertension management, community awareness campaigns including World Kidney Day activities, standardisation of the transition to renal replacement therapy, and strengthening of health literacy through patient education materials and digital health platforms [51]. Even though the programme has yet to be put into action, the CKD-AS-34 is well-positioned to serve as a monitoring and evaluation tool within this national programme framework, enabling identification of at-risk populations, targeting of interventions, and tracking of regional disparities in CKD awareness.

Furthermore, the relationship between scale results and demographic and clinical characteristics can be investigated to identify vulnerable populations and create culturally sensitive, individualised education programmes. The scale can be adapted for use in other stages of CKD, creating further opportunities for understanding and increasing CKD awareness. The scale’s demonstrated temporal stability further supports its use in longitudinal studies assessing intervention effectiveness.

### 4.5. Strengths and Limitations

This study’s strengths include a large, heterogeneous sample enabling robust psychometric analyses; a split-sample design preventing EFA-CFA cross-validation bias; comprehensive psychometric evaluation spanning multiple validity and reliability domains; strong theoretical grounding in established ecological frameworks; systematic scale development adhering to recommended guidelines; and incorporation of diverse expert perspectives, including patient representation.

However, several limitations merit consideration. First, the sample comprises stage 3–4 CKD patients from a single tertiary centre in Turkey; generalizability to other CKD stages, treatment modalities (dialysis, transplant), and cultural contexts necessitates validation studies. Furthermore, as a major tertiary centre, Ankara Etlik City Hospital serves patients with established nephrology follow-up, who may possess higher baseline awareness than community-dwelling patients with undiagnosed or primary-care-managed CKD. The scale’s performance in primary care, rural settings, and among patients not yet under specialist care remains to be established. Second, the cross-sectional design precludes causal inferences regarding awareness-outcome relationships. Third, the test–retest subsample (*n* = 30) is relatively modest, although ICC values are consistently excellent. Fourth, predictive validity linking awareness scores to clinical outcomes was not evaluated. Fifth, the scale requires cultural adaptation and validation prior to use in other countries and languages.

### 4.6. Future Research Directions

Several research avenues merit attention to further develop and extend the utility of the CKD-AS-34. Cross-cultural adaptation and validation in other countries and languages would enable international comparisons and establish the instrument’s generalisability beyond the Turkish context. Validation across other CKD stages and treatment modalities, including dialysis and post-transplant populations, would extend the scale’s applicability to the full disease trajectory, where awareness needs and challenges differ substantially. Prospective cohort studies examining the predictive relationships between CKD-AS-34 scores and clinical outcomes such as eGFR decline, emergency dialysis initiation, and hospitalisation would establish the instrument’s prognostic utility and inform clinically meaningful score thresholds for intervention targeting. Such thresholds could guide clinicians in identifying patients who would benefit most from specific awareness-enhancing interventions. The development of an abbreviated screening version, preserving the multidimensional structure while reducing respondent burden, could facilitate routine clinical use in busy outpatient settings. Finally, the scale’s sensitivity to change should be evaluated in intervention studies, including patient education programmes, digital health interventions, and community awareness campaigns, to determine whether the CKD-AS-34 can detect meaningful improvements in awareness following targeted interventions. Within Türkiye, the scale has immediate potential as a monitoring tool within the national Kidney Diseases Prevention and Control Programme (2025–2030), enabling systematic evaluation of awareness-related programme outcomes across regions.

## 5. Conclusions

This study developed an ecological model-based, multidimensional, reliable, and valid awareness scale for stage 3–4 CKD patients in Turkey. The CKD-AS-34, comprising thirty-four items and seven subdimensions, assesses awareness at Individual, Interpersonal/Institutional, Community, and Systemic levels alongside Treatment Adherence behaviours and Social Impact perceptions. The CKD-AS-34 demonstrated excellent factorial validity, strong internal consistency, excellent test–retest reliability, and meaningful associations with health literacy. This instrument addresses a critical methodological gap, presenting clinicians, researchers, and policymakers with a potential tool to assess patient awareness, guide targeted interventions, and monitor awareness programme effectiveness in the global endeavour to improve CKD outcomes.

## Figures and Tables

**Table 1:** Socio-demographic and clinical characteristics of the study population (*n* = 908).

Characteristic	Value (*n* = 908)
Age (years), mean ± SD (range)	57.6 ± 17.2 (18–94)
Sex, *n* (%)	
Male/Female	417 (45.9)/491 (54.1)
Marital status, *n* (%)	
Married/Single/Divorced/Widowed	588 (64.8)/126 (13.9)/43 (4.7)/146 (16.1)
Education level, *n* (%)	
Illiterate/Literate (no diploma)	10 (1.1)/154 (17.0)
Primary school/Middle school	292 (32.2)/94 (10.4)
High school/University or higher	177 (19.5)/182 (20.0)
Employment status, *n* (%)	
Employed/Retired/Unemployed	173 (19.1)/386 (42.5)/257 (28.3)
Unable to work (illness)/Student	77 (8.5)/16 (1.8)
Self-rated financial status (1–5), mean ± SD	3.00 ± 0.69
Comorbidities, *n* (%) †	
Hypertension	554 (61.0)
Diabetes mellitus	380 (41.9)
Cardiovascular Disease	297 (32.7)
Cerebrovascular Event	49 (5.4)

† Based on patient self-reports. Patients who reported uncertainty regarding their comorbidity status (10.6% for hypertension, 13.1% for diabetes, 13.1% for CVD, 20.9% for CVE) were classified as “unknown”.

**Table 2:** Reliability coefficients of the 41-item initial form subscales.

Subscale	Number of Items	Cronbach’s α	McDonald’s ω
Individual Awareness	8	0.833	0.855
Interpersonal Awareness	4	0.713	0.717
Institutional Awareness	4	0.559	0.628
Community Awareness	4	0.707	0.749
Systemic Awareness	4	0.663	0.672
Treatment Adherence	11	0.815	0.827
Social Impact (reverse-coded)	6	0.794	0.795
Ecological Model Total (q1–q24)	24	0.862	0.872
Total Scale (q1–q41)	41	0.895	0.900

**Table 3:** Eigenvalues and Explained Variance of Factors (36-Item EFA).

Factor	SS Loading	Explained Variance	Cumulative
F1 Individual Awareness	3.98	11.0%	11.0%
F2 Interpersonal/Institutional Awareness	2.72	7.6%	18.6%
F3 Social Impact	2.55	7.1%	25.7%
F4 Treatment Adherence I	2.11	5.9%	31.5%
F5 Treatment Adherence II	1.50	4.2%	35.7%
F6 Community Awareness	1.66	4.6%	40.3%
F7 Systemic Awareness	1.24	3.4%	43.8%

**Table 4:** CFA Model Comparison.

Model	χ^2^	df	CFI	TLI	RMSEA (90% CI)	SRMR
36 Items	2059.4	573	0.966	0.962	0.076 (0.072–0.079)	0.078
35 Items	1807.7	539	0.969	0.966	0.072 (0.068–0.076)	0.076
34 Items (Final)	1642.4	506	0.972	0.969	0.070 (0.067–0.074)	0.075

**Table 5:** Average Variance Extracted (AVE) and Composite Reliability (CR) values.

Factor	Number of Items	AVE	CR
Individual Awareness	6	0.772	0.953
Interpersonal/Institutional Awareness	8	0.453	0.868
Community Awareness	3	0.656	0.850
Systemic Awareness	4	0.417	0.740
Treatment Adherence I	4	0.644	0.878
Treatment Adherence II	4	0.451	0.767
Social Impact	5	0.465	0.811

**Table 6:** Factor correlation matrix (√AVE on diagonal).

	Individual	Inter/Int	Social Imp	TA I	TA II	Community	Systemic
Individual Awareness	0.879	0.388	0.219	0.203	0.260	0.167	0.332
Interpersonal/ Institutional Awareness	0.388	0.673	0.331	0.369	0.394	0.112	0.512
Social Impact	0.219	0.331	0.682	0.320	0.684	0.314	0.422
Treatment Adherence I	0.203	0.369	0.320	0.803	0.538	0.122	0.215
Treatment Adherence II	0.260	0.394	0.684	0.538	0.672	0.180	0.406
Community Awareness	0.167	0.112	0.314	0.122	0.180	0.810	0.562
Systemic Awareness	0.332	0.512	0.422	0.215	0.406	0.562	0.646

Diagonal values represent √AVE; off-diagonal values represent inter-factor correlations.

**Table 7:** Internal consistency reliability coefficients of the final 34-item model (*n* = 908).

Subscale	Number of Items	Cronbach’s α	McDonald’s ω
Individual Awareness	6	0.891	0.901
Interpersonal/Institutional Awareness	8	0.808	0.816
Community Awareness	3	0.735	0.765
Systemic Awareness	4	0.660	0.669
Treatment Adherence I	4	0.821	0.829
Treatment Adherence II	4	0.656	0.697
Social Impact	5	0.783	0.784
Total Scale	34	0.884	0.889

**Table 8:** Test–retest reliability coefficients (*n* = 30).

Subscale	Number of Items	Pearson r	ICC(3, 1)	95% CI
Individual Awareness	6	0.980	0.964	0.927–0.983
Interpersonal/Institutional Awareness	8	0.980	0.972	0.942–0.987
Community Awareness	3	0.930	0.915	0.830–0.959
Systemic Awareness	4	0.944	0.904	0.808–0.953
Treatment Adherence I	4	0.955	0.936	0.871–0.969
Treatment Adherence II	4	0.932	0.883	0.768–0.942
Social Impact	5	0.955	0.931	0.859–0.966
Total Scale	34	0.976	0.954	0.907–0.978

## Data Availability

The datasets used during the study have not been made publicly available due to patient privacy but are available from the corresponding author upon reasonable request.

## Supplementary Materials

The following supporting information can be downloaded at: https://www.mdpi.com/article/10.3390/healthcare14070876/s1. The validated Turkish Scale, the English translation (unvalidated; for research purposes) and the scoring material can be found in the Supplementary Materials.

## Abbreviations

The following abbreviations are used in this manuscript:

AVE	Average Variance Extracted
CFA	Confirmatory Factor Analysis
CFI	Comparative Fit Index
CI	Confidence Interval
CKD	Chronic Kidney Disease
CKD-AS-34	Chronic Kidney Disease Awareness Scale-34
CR	Composite Reliability
CREDIT	Chronic REnal Disease in Turkey
CVI	Content Validity Index
DWLS	Diagonally Weighted Least Squares
EFA	Exploratory Factor Analysis
GBD	Global Burden of Disease
h^2^	Communality
ICC	Intraclass Correlation Coefficient
IQR	Interquartile Range
KDIGO	Kidney Disease: Improving Global Outcomes
KMO	Kaiser-Meyer-Olkin
minres	Minimum Residual
MSA	Measure of Sampling Adequacy
OR	Odds Ratio
REVEAL-CKD	Real-world Evidence for VALidation of Chronic Kidney Disease
RMSEA	Root Mean Square Error of Approximation
RMSR	Root Mean Square Residual
RRT	Renal Replacement Therapy
SD	Standard Deviation
SRMR	Standardised Root Mean Square Residual
TLI	Tucker–Lewis Index
TSOY-32	Türkiye Sağlık Okuryazarlığı Ölçeği-32 (Turkish Health Literacy Scale-32)
WHO	World Health Organisation
α	Cronbach’s Alpha
β	Standardised Beta Coefficient
λ	Factor Loading
ω	McDonald’s Omega
χ^2^	Chi-Square
