# Supplementary material for: Development and Psychometric Validation of a Multidimensional Ecological Model-Based Awareness Scale for Patients with Stage 3–4 Chronic Kidney Disease
Source: Healthcare (Basel). 2026 Mar 28;14(7):876. doi: 10.3390/healthcare14070876 (PMC13072799; doi:10.3390/healthcare14070876)
Supplement: Supplementary file 1 [file healthcare-14-00876-s001.zip › supplements ckd copy.pdf]

Development And Psychometric Validation Of A Multidimensional Ecological Model-Based Awareness Scale for Patients with Stage 3-4 Chronic Kidney Disease

Supplementary Materials

Supplementary Table S1. *Descriptive Statistics and Item Characteristics (41 Items)*

Item	Subscale	Mean + SD	Median	Skewness	Kurtosis	Floor (%)	Ceiling (%)
q1	Individual	3.24±1.22	2	0.12	-1.14	10.4	12.1
q2	Individual	1.91±1.09	1	0.81	-0.61	51.8	0.99
q3	Individual	2.99±1.08	3	-0.34	-0.66	3.7	13.5
q4	Individual	4.13±1.03	4	-1.42	1.48	1.0	46.0
q5	Individual	3.48±1.19	4	-0.63	-0.46	3.9	16.3
q6	Individual	2.26±0.82	2	0.46	0.25	2.8	0.09
q7	Individual	1.44±0.69	1	1.54	2.04	65.7	0.11
q8	Individual	3.31±0.84	3	-0.11	-0.37	0.8	0.09
q9	Interpersonal	3.49±1.15	4	-0.76	-0.26	3.9	15.9
q10	Interpersonal	3.94±0.97	4	-1.15	1.35	0.3	28.1
q11	Interpersonal	4.00±0.99	4	-1.10	0.93	0.3	34.1
q12	Interpersonal	3.54±1.13	4	-0.72	-0.26	3.9	17.8
q13	Institutional	4.06±0.88	4	-1.19	1.88	0.3	31.2
q14	Institutional	4.16±0.90	4	-1.31	1.91	0.3	40.5
q15	Institutional	3.79±1.21	4	-0.94	0.04	0.4	32.8
q16	Institutional	2.98±0.80	3	0.05	-0.19	0.2	0.00
q17	Community	2.09±1.10	2	0.61	-0.63	3.7	2.09
q18	Community	2.64±1.25	3	0.10	-1.22	0.4	0.51
q19	Community	1.60±0.79	1	1.27	1.41	54.6	0.33
q20	Community	1.69±0.89	1	1.44	1.99	52.2	1.32
q21	Systemic	3.48±1.17	4	-0.69	-0.27	2.9	18.2
q22	Systemic	3.86±1.05	4	-1.01	0.68	0.3	28.5
q23	Systemic	2.82±1.27	3	0.01	-1.08	4.2	12.6
q24	Systemic	2.91±1.29	3	0.04	-1.26	4.9	12.7
q25	Treatment Adherence	3.17±1.21	3	-0.13	-0.94	4.0	15.7

q26*	Treatment Adherence	3.34±1.29	3	-0.29	-1.03	6.6	23.2
q27	Treatment Adherence	3.72±1.18	4	-0.64	-0.54	3.9	32.3
q28	Treatment Adherence	2.92±1.16	3	0.01	-0.75	3.8	13.2
q29	Treatment Adherence	2.97±1.14	3	-0.01	-0.72	3.8	11.1
q30	Treatment Adherence	4.41±0.85	5	-1.71	3.16	1.5	57.8
q31	Treatment Adherence	2.92±1.23	3	0.09	-0.79	3.7	10.0
q32	Treatment Adherence	3.91±1.05	4	-0.93	0.38	0.4	32.8
q33	Treatment Adherence	3.29±1.24	4	-0.27	-1.44	5.1	19.9
q34	Treatment Adherence	2.96±1.14	3	0.07	-1.29	4.6	20.2
q35	Treatment Adherence	4.18±1.02	4	-1.30	1.27	0.3	48.1
q36†	Social Impact	3.35±1.19	3	-0.30	-0.81	4.9	16.1
q37†	Social Impact	3.06±1.47	3	-0.07	-1.39	4.9	23.5
q38†	Social Impact	3.68±1.21	4	-0.63	-0.57	4.0	31.7
q39†	Social Impact	3.30±1.38	3	-0.26	-1.16	4.5	26.2
q40†	Social Impact	3.07±1.35	3	-0.04	-1.17	4.3	19.9
q41†	Social Impact	3.13±1.27	3	-0.07	-1.01	4.2	18.5

**q26: only applied patients with diabetes(n=380)*

†q36-q41: reverse-point system (6 minus the original item score).

Floor (%):Percentage of the lowest score category (1) among all answers

Ceiling (%): Percentage of the highest score category (5) in all answers

Supplementary Table S2. EFA Item Summary: Factor Assignment, Primary Loadings, Communalities, and Retention Status (41-Item Initial Pool, n=454)

Item	Factor	Primary λ	h^2	u^2	Com	Status
**q1**	Individual	**0.86**	0.742	0.258	1.02	Retained
**q2**	Individual	**0.73**	0.640	0.360	1.16	Retained
**q3**	Individual	0.39	0.301	0.699	1.99	*Removed (CFA: cross-loading)*
**q4**	Inter/Inst	**0.49**	0.372	0.628	2.14	Retained
**q5**	Inter/Inst	**0.40**	0.347	0.653	2.60	Retained
**q6**	Individual	**0.87**	0.785	0.215	1.03	Retained
**q7**	Individual	**0.74**	0.562	0.438	1.04	Retained
**q8**	Individual	**0.66**	0.426	0.574	1.10	Retained
**q9**	Inter/Inst	**0.48**	0.294	0.706	1.23	Retained
**q10**	Inter/Inst	**0.69**	0.536	0.464	1.18	Retained
**q11**	Inter/Inst	**0.60**	0.411	0.589	1.31	Retained
**q12**	Inter/Inst	**0.52**	0.400	0.600	1.63	Retained
**q13**	Inter/Inst	**0.66**	0.480	0.520	1.10	Retained
**q14**	Inter/Inst	**0.69**	0.534	0.466	1.16	Retained
**q15**	—	0.30	0.135	0.865	2.31	*Removed (EFA-1: h^2=0.14)*
**q16**	Individual	**0.78**	0.635	0.365	1.03	Retained
**q17**	Community	**0.57**	0.446	0.554	1.36	Retained
**q18**	Systemic	0.32	0.318	0.682	3.20	*Removed (EFA-2: high complexity)*
**q19**	Community	**0.76**	0.605	0.395	1.02	Retained
**q20**	Community	**0.66**	0.484	0.516	1.10	Retained
**q21**	Systemic	**0.53**	0.402	0.598	1.30	Retained
**q22**	Systemic	**0.48**	0.323	0.677	1.75	Retained
**q23**	Systemic	**0.57**	0.497	0.503	1.44	Retained
**q24**	Systemic	**0.33**	0.226	0.774	1.88	Retained
**q25**	TA-I	0.30	0.216	0.784	2.25	*Removed (EFA-2: h^2<0.20)*

**q26**	TA-I	0.37	0.171	0.829	2.48	*Removed (EFA-1: h^2=0.17)*
**q27**	TA-I	**0.52**	0.395	0.605	1.33	Retained
**q28**	TA-I	**0.82**	0.656	0.344	1.01	Retained
**q29**	TA-I	**0.81**	0.660	0.340	1.03	Retained
**q30**	TA-II	**0.32**	0.350	0.650	3.65	Retained
**q31**	TA-I	**0.59**	0.479	0.521	1.20	Retained
**q32**	TA-II	**0.36**	0.414	0.586	2.68	Retained
**q33**	TA-II	**0.59**	0.479	0.521	1.18	Retained
**q34**	TA-II	0.48	0.477	0.523	1.68	*Removed (EFA-2: λ<0.30 in 39-item)*
**q35**	TA-II	**0.33**	0.379	0.621	3.51	Retained
**q36**	Social Impact	**0.48**	0.378	0.622	1.29	Retained
**q37**	Social Impact	**0.63**	0.494	0.506	1.19	Retained
**q38**	Social Impact	**0.36**	0.462	0.538	2.44	Retained
**q39**	Social Impact	**0.70**	0.547	0.453	1.06	Retained
**q40**	Social Impact	**0.76**	0.602	0.398	1.04	Retained
**q41**	Social Impact	0.66	0.360	0.640	1.14	*Removed (CFA: λ=0.449)*

Note: *EFA conducted using minimum residual extraction with oblimin rotation (7-factor solution). Primary λ = highest factor loading for each item. h^2 = communality; u^2 = uniqueness; Com = Hofmann complexity. Red-shaded rows: items removed during iterative refinement. EFA-1 = first iteration (41→39 items); EFA-2 = second iteration (39→36 items); CFA = confirmatory phase (36→34 items). Pre-specified removal criteria: $h^2 < 0.20$, $\lambda < 0.30$, or complexity > 2.0 (Tabachnick & Fidell, 2019).*

Supplementary Table S3. Parallel Analysis: Observed vs. Simulated Eigenvalues (n=454, 41 items, 1000 Monte Carlo iterations)

Factor	Observed Eigenvalue	Simulated Mean Eigenvalue	Simulated 95th Percentile	Retain? (PA Criterion)
**1**	8.623	1.622	1.693	YES
**2**	3.495	1.552	1.608	YES
**3**	2.968	1.500	1.543	YES
**4**	2.376	1.454	1.493	YES
**5**	1.972	1.414	1.448	YES
**6**	1.442	1.377	1.409	YES
**7**	1.227	1.343	1.374	NO*
**8**	1.160	1.310	1.340	NO
**9**	1.050	1.280	1.310	NO
**10**	1.008	1.249	1.278	NO

*Parallel analysis compares observed eigenvalues with eigenvalues generated from random data of equal dimensions. Factors are retained when the observed eigenvalue exceeds the simulated 95th percentile. Parallel analysis supported a six-factor solution. *Factor 7 (observed eigenvalue = 1.227) fell below the simulated 95th percentile (1.374) but exceeded the Kaiser criterion (eigenvalue > 1.0). The seven-factor solution was retained based on: (1) superior model fit compared to the six-factor solution (TLI: 0.863 vs. 0.846; RMSEA: 0.049 vs. 0.052); (2) theoretical alignment with the socioecological model framework; and (3) improved interpretability of factor structure. This decision process is consistent with recommendations to integrate multiple criteria for factor retention rather than relying on any single method.*

Supplementary Table S4. Standardized Factor Loadings of the 34-Item Final Model

Factor	Item	Std. Loading
**Individual Awareness**	q1	0.886
	q2	0.803
	q6	0.939
	q7	0.923
	q8	0.738
	q16	0.962
**Interpersonal/Institutional**	q4	0.538
	q5	0.647
	q9	0.719
	q10	0.720
	q11	0.666
	q12	0.604
	q13	0.711
	q14	0.755
**Community Awareness**	q17	0.701
	q19	0.894
	q20	0.823
**Systemic Awareness**	q21	0.701
	q22	0.691
	q23	0.610
	q24	0.572
**Treatment Adherence I**	q27	0.687
	q28	0.845
	q29	0.901
	q31	0.760
**Treatment Adherence II**	q30	0.676
	q32	0.708
	q33	0.645
	q35	0.657
**Social Impact**	q36	0.621
	q37	0.777

	q38	0.740
	q39	0.654
	q40	0.600

Supplementary Table S5. Fit Indices Comparison of Alternative Models

Model	CFI	TLI	RMSEA	SRMR
**Empirical 7-Factor (Final)**	0.972	0.969	0.070	0.075
**Theoretical 7-Factor (improved)**	0.945	0.939	0.099	0.097
**Theoretical 7-Factor (raw)**	0.928	0.920	0.114	0.109
**5-Factor Ecological (improved)**	0.924	0.917	0.115	0.112
**5-Factor Ecological (raw)**	0.908	0.901	0.127	0.120
**3-Factor Ecological**	0.859	0.849	0.156	0.138
**Single-Factor**	0.776	0.761	0.196	0.165

Model specifications:

Empirical 7-Factor (Final): *Factor structure as determined by EFA and confirmed by CFA (see Section 3.5 for item assignments).*

Theoretical 7-Factor: *Items assigned to original a priori subscales: Individual (q1–q8), Interpersonal (q9–q12), Institutional (q13–q16), Community (q17–q20), Systemic (q21–q24), Treatment Adherence (q27–q31, q33, q35), Social Impact (q36–q40). This model maintains Interpersonal and Institutional as separate factors and treats Treatment Adherence as a single factor. "Improved" version excludes the two items removed during CFA (q3, q41); "raw" version includes all 36 EFA-retained items.*

5-Factor *Ecological: Individual (q1, q2, q6–q8, q16), Interpersonal/Institutional combined (q4, q5, q9–q14), Community/Systemic combined (q17, q19–q24), Treatment Adherence combined (q27–q31, q33, q35), Social Impact (q36–q40). "Improved" excludes q3 and q41; "raw" includes 36 items.*

3-Factor Ecological: *Proximal Awareness (all Individual + Interpersonal/Institutional items), Distal Awareness (Community + Systemic), Behavioural (all Treatment Adherence items+ Social Impact items).*

Single-Factor: *All 34 items loading onto a single latent factor.*

Supplementary Table S6. Kronik Böbrek Hastalığı Farkındalık Ölçeği (KBH-FÖ-34) / ***Chronic Kidney Disease Awareness Scale (CKD-AS-34) - Validated Turkish Version***

Madde	Hiç Katılmıyorum	Katılmıyorum	Kararsızım	Katılıyorum	Tamamen Katılıyorum
Bireysel Farkındalık (6 madde)					
1. Kronik böbrek hastalığının belirtileri ve ilerleyişi hakkında bilgi sahibiyim.	☐	☐	☐	☐	☐
2. Glomerüler filtrasyon hızı (GFH/GFR) kavramının ne anlama geldiğini biliyorum.	☐	☐	☐	☐	☐
3. Hemodiyaliz ne anlama geliyor biliyorum.	☐	☐	☐	☐	☐
4. Periton diyalizi ne anlama geliyor biliyorum.	☐	☐	☐	☐	☐
5. Böbrek nakli ne anlama geliyor biliyorum.	☐	☐	☐	☐	☐
6. Böbrek hastalığım ilerlerse diyalize veya böbrek nakline ihtiyacım olabileceğini biliyorum.	☐	☐	☐	☐	☐
Kişilerarası/Örgütsel Farkındalık (8 madde)					
7. Kronik böbrek hastalığının ilerleyici bir hastalık olduğunu biliyorum.	☐	☐	☐	☐	☐
8. Böbrek sağlığımı korumak için yaşam tarzımı nasıl düzenlemem gerektiğini biliyorum.	☐	☐	☐	☐	☐
9. Ailem hastalığım ile ilgili ihtiyaçlarıma ve sorunlarıma saygı gösterir	☐	☐	☐	☐	☐
10. Ailem, ilaçlarımı düzenli kullanmam için bana destek olur	☐	☐	☐	☐	☐
11. Romantik ilişkilerde (eş, sevgili, partner) hastalığım nedeni ile zorluk yaşamıyorum.	☐	☐	☐	☐	☐
12. Nefroloji polikliniğinde bana verilen bilgilerden fayda gördüm.	☐	☐	☐	☐	☐
13. Sağlık ekibim ile iyi bir iletişimim var.	☐	☐	☐	☐	☐
14. Doktorumdan aldığım bilgiler hastalığımı anlamamı kolaylaştırdı	☐	☐	☐	☐	☐
Toplumsal Farkındalık (3 madde)					

15. Toplumda böbrek sağlığına yönelik genel farkındalık yeterlidir.	☐	☐	☐	☐	☐
16. Sosyal çevremde farkındalığı arttıran etkinlikler düzenleniyor.	☐	☐	☐	☐	☐
17. Diğer böbrek hastaları ile deneyimlerimi paylaşabileceğim ortamlar var.	☐	☐	☐	☐	☐
Sistemsel Farkındalık (4 madde)					
18. Devlet hastanesinde nefroloji takibine erişmem kolaydır.	☐	☐	☐	☐	☐
19. Reçeteli ilaçların fiyatlandırması tedaviye erişimimi zorlaştırmıyor.	☐	☐	☐	☐	☐
20. Sağlık sistemindeki bilgilendirme politikalarını yeterli buluyorum.	☐	☐	☐	☐	☐
21. Sağlık tesislerine ulaşım hizmetlerini (dolmuş, otobüs, park yeri) yeterli buluyorum.	☐	☐	☐	☐	☐
Tedavi Uyumu I (4 madde)					
	**Hiçbir Zaman**	**Nadiren**	**Bazen**	**Sıklıkla**	**Her Zaman**
22. Günlük tuz tüketimime dikkat ederim.	☐	☐	☐	☐	☐
23. Düşük proteinli diyet uygulamaya çalışırım.	☐	☐	☐	☐	☐
24. Yüksek potasyum içeren gıdalardan kaçınırım.	☐	☐	☐	☐	☐
25. Yüksek fosfor içeren gıdalardan kaçınırım	☐	☐	☐	☐	☐
Tedavi Uyumu II (4 madde)					
26. İlaçlarımı düzenli şekilde kullanırım.	☐	☐	☐	☐	☐
27. Önerilen sıvı miktarlarında tüketim yapmaya dikkat ederim.	☐	☐	☐	☐	☐
28. Gereksiz ilaç kullanmaktan kaçınırım.	☐	☐	☐	☐	☐
29. Doktor kontrollerime düzenli giderim.	☐	☐	☐	☐	☐
Sosyal Etki (5 madde)					
30. Beslenme kısıtlamalarım sosyal hayatıma engel oluyor.	☐	☐	☐	☐	☐
31. Hastalığım seyahat etmeme engel oluyor.	☐	☐	☐	☐	☐

32. Hastalığım nedeni ile sosyal yaşantımda yargılandığımı hissediyorum.	☐	☐	☐	☐	☐
33. Hastalığım nedeni ile sosyal etkinliklere katılmaktan kaçınıyorum.	☐	☐	☐	☐	☐
34. Hastalığım nedeni ile (tedavi masrafları, hastalık sebebi ile çalışamama) maddi sorun yaşıyorum.	☐	☐	☐	☐	☐
**Puanlama:** Maddeler 1-21 için: Hiç Katılmıyorum=1, Katılmıyorum=2, Kararsızım=3, Katılıyorum=4, Tamamen Katılıyorum=5. Maddeler 22-34 için: Hiçbir Zaman=1, Nadiren=2, Bazen=3, Sıklıkla=4, Her Zaman=5.<br>**Ters Puanlanan Maddeler:** Sosyal Etki alt boyutu maddeleri (30-34) ters puanlanır.					

This table presents the original validated Turkish version of the CKD-AS-34. An English translation is provided in Table S7.

Supplementary Table S7. Chronic Kidney Disease Awareness Scale (CKD-AS-34) - ***English Translation (for reference purposes – not validated)***

Item	Strongly Disagree	Disagree	Neutral	Agree	Strongly Agree
Individual Awareness (6 items)					
1. I know about the symptoms and progression of chronic kidney disease.	☐	☐	☐	☐	☐
2. I understand what glomerular filtration rate (GFR) means.	☐	☐	☐	☐	☐
3. I understand what haemodialysis means.	☐	☐	☐	☐	☐
4. I understand what peritoneal dialysis means.	☐	☐	☐	☐	☐
5. I understand what kidney transplantation means.	☐	☐	☐	☐	☐
6. I understand that I might need dialysis or transplantation if my disease progresses.	☐	☐	☐	☐	☐
Interpersonal/Institutional Awareness (8 items)					
7. I understand that Chronic Kidney Disease is progressive.	☐	☐	☐	☐	☐
8. I know how to adjust my lifestyle to protect my kidney health	☐	☐	☐	☐	☐
9. My family respects my needs and concerns related to my disease.	☐	☐	☐	☐	☐
10. My family supports me in taking my medications regularly.	☐	☐	☐	☐	☐
11. I do not experience difficulties in romantic relationships (spouse, partner) due to my disease.	☐	☐	☐	☐	☐
12. I have benefited from the information provided at the nephrology outpatient clinic.	☐	☐	☐	☐	☐
13. I have good communication with my healthcare team.	☐	☐	☐	☐	☐
14. The information I received from my doctor helped me understand my disease.	☐	☐	☐	☐	☐
Community Awareness (3 items)					
15. General awareness of kidney health in society is adequate.	☐	☐	☐	☐	☐
16. Awareness-raising activities are organised in my social environment.	☐	☐	☐	☐	☐
17. There are settings where I can share my experiences with other kidney patients.	☐	☐	☐	☐	☐
Systemic Awareness (4 items)					

18. It is easy for me to access nephrology follow-up at a public hospital.	☐	☐	☐	☐	☐
19. The pricing of prescription medications does not hinder my access to treatment.	☐	☐	☐	☐	☐
20. I find the health information policies within the healthcare system adequate.	☐	☐	☐	☐	☐
21. I find the transportation services to healthcare facilities (public transport, parking) adequate.	☐	☐	☐	☐	☐
Treatment Adherence I (4 items)					
	**Never**	**Rarely**	**Sometimes**	**Often**	**Always**
22. I pay attention to my daily salt intake.	☐	☐	☐	☐	☐
23. I try to follow a low-protein diet.	☐	☐	☐	☐	☐
24. I avoid foods high in potassium.	☐	☐	☐	☐	☐
25. I avoid foods high in phosphorus	☐	☐	☐	☐	☐
Treatment Adherence II (4 items)					
26. I take my medications regularly.	☐	☐	☐	☐	☐
27. I pay attention to consuming the recommended amount of fluids.	☐	☐	☐	☐	☐
28. I avoid unnecessary medications	☐	☐	☐	☐	☐
29. I attend my doctor appointments regularly.	☐	☐	☐	☐	☐
Social Impact (5 items)					
30. My dietary restrictions interfere with my social life.	☐	☐	☐	☐	☐
31. My disease prevents me from travelling.	☐	☐	☐	☐	☐
32. I feel judged in my social life because of my disease.	☐	☐	☐	☐	☐
33. I avoid participating in social activities because of my disease.	☐	☐	☐	☐	☐
34. I experience financial difficulties due to my disease (treatment costs, inability to work due to illness).	☐	☐	☐	☐	☐

Scoring: For items 1-21: Strongly Disagree=1, Disagree=2, Neutral=3, Agree=4, Strongly Agree=5. For items 22-34: Never=1, Rarely=2, Sometimes=3, Often=4, Always=5.

Reverse-Scored Items: Social Impact subscale items (30-34) are reverse-scored.

Note: *This English version is provided for reference purposes. The validated scale was developed and tested in Turkish. Cross-cultural adaptation and validation studies are required before use in English-speaking populations.*
